# Acrylate-Based Hybrid Sol-Gel Coating for Corrosion Protection of AA7075-T6 in Aircraft Applications: The Effect of Copolymerization Time

**DOI:** 10.3390/polym12040948

**Published:** 2020-04-19

**Authors:** Peter Rodič, Romana Cerc Korošec, Barbara Kapun, Alenka Mertelj, Ingrid Milošev

**Affiliations:** 1 Department of Physical and Organic Chemistry, Jožef Stefan Institute, Jamova c. 39, SI-1000 Ljubljana, Slovenia; barbara.kapun@ijs.si (B.K.); ingrid.milosev@ijs.si (I.M.); 2 Faculty of Chemistry and Chemical Technology, University of Ljubljana, Večna pot 113, SI-1000 Ljubljana, Slovenia; romana.cerc-korosec@fkkt.uni-lj.si; 3 Department of Complex Matter, Jožef Stefan Institute, Jamova c. 39, SI-1000 Ljubljana, Slovenia; alenka.mertelj@ijs.si

**Keywords:** AA7075-T6, hybrid sol-gel, copolymerization, corrosion, dilute Harrison’s solution

## Abstract

Pre-hydrolysed/condensed tetraethyl orthosilicate (TEOS) was added to a solution of methyl methacrylate (MMA) and 3-methacryloxypropyltrimethoxysilane (MAPTMS), and then copolymerised for various times to study the influence of the latter on the structure of hybrid sol-gel coatings as corrosion protection of aluminium alloy 7075-T6. The reactions taking place during preparation were characterised using real-time Fourier transform infrared spectroscopy, dynamic light scattering and gel permeation chromatography. The solution characteristics were evaluated, using viscosimetry, followed by measurements of thermal stability determined by thermogravimetric analysis. The optimal temperature for the condensation reaction was determined with the help of high-pressure differential scanning calorimetry. Once deposited on 7075-T6 substrates, the coatings were evaluated using a field emission scanning electron microscope coupled to an energy dispersive spectrometer to determine surface morphology, topography, composition and coating thickness. Corrosion properties were tested in dilute Harrison’s solution (3.5 g/L (NH_4_)_2_SO_4_ and 0.5 g/L NaCl) using electrochemical impedance spectroscopy. The copolymerization of MMA and MAPTMS over 4 h was optimal for obtaining 1.4 µm thick coating with superior barrier protection against corrosion attack (|Z_10 mHz_| ~ 1 GΩ cm^2^) during three months of exposure to the corrosive medium.

## 1. Introduction

Aluminium alloy (AA) 7075-T6, with Zn, Mg and Cu as major alloying elements, has excellent physical properties [1], such as low density and high strength. It is often used in the aircraft industry as fuselage skin and wing spars [2]. However, due to the presence of alloying elements that form intermetallic particles [1,3,4,5,6,7], the alloy has a low resistance to corrosion when exposed to chloride [3,4,5,8] and other ions, such as sulphates, present in the atmosphere environment [9,10,11]. Corrosion, induced by these ions, particularly at the intermetallic particles, can damage the structural parts of the aeroplane, causing mechanical failure [12].

The state-of-the-art in corrosion protection in the aerospace industry is comprised of a multi-layer system consisting of pre-treatment, primer and topcoat [13]. These components should exhibit proper functionality, presumably also active corrosion protection that is able to prolong the lifespan of the device, part or construction made of aluminium alloys [14,15,16]. Pre-treatment and primer are usually based on chromates [17,18] but, due to the hazardous impact of Cr(VI) species on the environment [19] and on human health [20], several restrictions and regulations have been accepted worldwide.

Nowadays, there is an increase in technological and economic requirements for protecting aluminium alloys in aggressive environments [21]. Organic–inorganic hybrid sol-gel coatings have been recognised as prospective candidates for the substitution of chromate-based coatings [14,16,22,23]. Hybrid coatings combine the functional qualities of organic and inorganic materials [24,25], since the paths of synthesis offer the possibility of precise control of the reaction parameters and of incorporating various organic monomers so that the coating properties can be varied extensively. One of the commonly used organic monomers is methyl methacrylate (MMA), which is a low-cost, non-toxic, transparent and colourless polymer, commonly used for various purposes, from optic lenses to protective coatings and as an alternative to glass [26,27]. Due to the presence of silanol (Si–OH) groups originating from organically-modified silicon alkoxides (silanes) (such as 3-methacryloxypropyltrimethoxysilane, MAPTMS) and inorganic silicon alkoxides (such as tetraethyl orthosilicate, TEOS), hybrid materials have excellent adhesion on various aluminium substrates with Al–OH groups on the surface [28,29,30,31].

Studies of poly(methyl methacrylate) (PMMA) have reported a variety of synthesis conditions, differing in MMA/MAPTMS ratio, the ethanol-to-water ratio in the inorganic phase [24,27,32,33,34], synthesis temperature [27] and atmosphere conditions [33,35,36,37,38]. PMMA coatings have been deposited on a variety of substrates and have been used for barrier corrosion protection on AA2024-T3 [32,37,38] and steel [27,33,39,40].

In general, the copolymerization process in the synthesis is based on the reaction between the acrylate group of organic monomer and organically modified silicon alkoxide containing an alkyl tail with an acrylate functional group, forming a network in the presence of coupling agent [24,35,36,37,38,41]. In the presence of acidic water, the hydrolysis/condensation reactions of silicon alkoxide groups also occur, resulting in the formation of a Si−O−Si (siloxane) network. It is known that well-controlled copolymerization conditions are crucial for obtaining a polymer with desired properties [27,32,35,36]. The copolymerization can be stimulated using various initiators; the most frequently used are free-radical polymerisation initiators, which give a high degree of C=C conversion such as benzoyl peroxide (BPO) [42,43] as a thermally induced initiator and 2,2′-azobis(2-methylpropionitrile) (AIBN) [32,42,43,44] as a thermally/UV induced initiator. The degree of copolymerization involves three time-dependent steps: initiation, propagation and polymerisation [35,38,45]. Once the copolymerization process is stopped at a certain level, further hydrolysis and condensation can continue, based on the sol-gel approach, in the absence or presence of another silane, i.e., TEOS [27,29,37,38].

In the previous studies on the synthesis of hybrid sol-gel coatings, the focus was mainly on the hydrolysis and condensation reactions, forming the organically modified silicates (ORMOSILS) [27,29,34,39,40,46,47,48,49] or other organically modified ceramics (ORMOCERS) [50,51,52]. The correlation between the degree of polymerisation of functional groups of organically modified silanes and additional monomers in the sol has been less investigated, especially the effect on the coating properties. Higher degrees of copolymerization, which can be achieved by the addition of cerium nitrate, have been shown to enhance the corrosion barrier properties [53,54,55,56]. In our previous study, it was shown that the type of atmosphere (air or nitrogen) affects the degree of copolymerization of MMA and MAPTMS [38]. The acrylate-based coatings, synthesised under nitrogen atmosphere, provided excellent barrier protection for AA2024-T3 for more than six months in 0.1 M NaCl, with impedance values of up to 1 GΩ cm^2^, and durable corrosion performance of up to 500 h in a salt spray chamber [38]. Optimised copolymerization conditions therefore have an important impact on coating properties and should be studied further in more detail.

Considering the great potential of poly(methyl methacrylate) for various applications, we focused on the characterisation of each step of the synthesis to obtain high-yield copolymerization during well-controlled preparation. The system investigated consisted of TEOS as the inorganic silane and MMA and MAPTMS as the organic monomer and the organically modified silane. We targeted copolymerization time as an important parameter that controls the physical and chemical properties and, consequently, the corrosion performance of the coatings. The synthesis process was followed using real-time Fourier transform infrared (FTIR) spectroscopy, dynamic light scattering (DLS) and gel permeation chromatography (GPC). Solution properties were evaluated using viscosity and density measurements. Thermogravimetric characterisation was also performed. The coating properties were evaluated using field emission scanning electron microscopy coupled to an energy dispersive spectrometer (FESEM/EDS) to determine coating homogeneity, structure, composition and morphology. The corrosion properties were evaluated using electrochemical impedance spectroscopy (EIS) during immersion in dilute Harrison’s solution, which simulates aircraft conditions.

## 2. Materials and Methods

### 2.1. Chemicals for Solution Preparation

Tetraethyl orthosilicate (TEOS: Si(OC_2_H_5_)_4_, 99.9%, Aldrich, Munich, Germany, CAS Number: 78-10-4) was hydrolysed with deionised water Milli-Q Direct, resistivity 18.2 MΩ cm at 25 °C (Millipore, Billerica, MA) and nitric acid (HNO_3_: >70%, Sigma-Aldrich, Milano, Italy, CAS Number: 7697-37-2). Other chemicals used in the synthesis were methyl methacrylate (MMA: CH_2_=C(CH_3_)CO_2_CH_3_, which contains ≤30 ppm 4-methoxyphenol (MEHQ: C_7_H_8_O_2_) as polymerisation inhibitor, 99.0% Aldrich, Munich, Germany, CAS Number 80-62-6), and 3-methacryloxypropyltrimethoxysilane (MAPTMS: H_2_C=C(CH_3_)CO_2_(CH_2_)_3_Si(OCH_3_)_3_, ≥98% Sigma, St. Louis, MO, USA, CAS Number: 2530-85-0). The benzoyl peroxide (BPO: (C_6_H_5_CO)_2_O_2_, Aldrich, Munich, Germany, CAS Number: 94-36-0) was added as a radical initiator. The reaction was performed in tetrahydrofuran (THF: (CH_2_)_4_O, anhydrous, ≥99.9%, inhibitor-free Sigma-Aldrich, Munich, Germany, CAS Number: 109-99-9) as a solvent. Chemicals were used as received without purification; the only exception was MMA which was distilled three times at low vacuum to remove MEHQ.

### 2.2. Synthesis of the Solution

**Figure 1:**
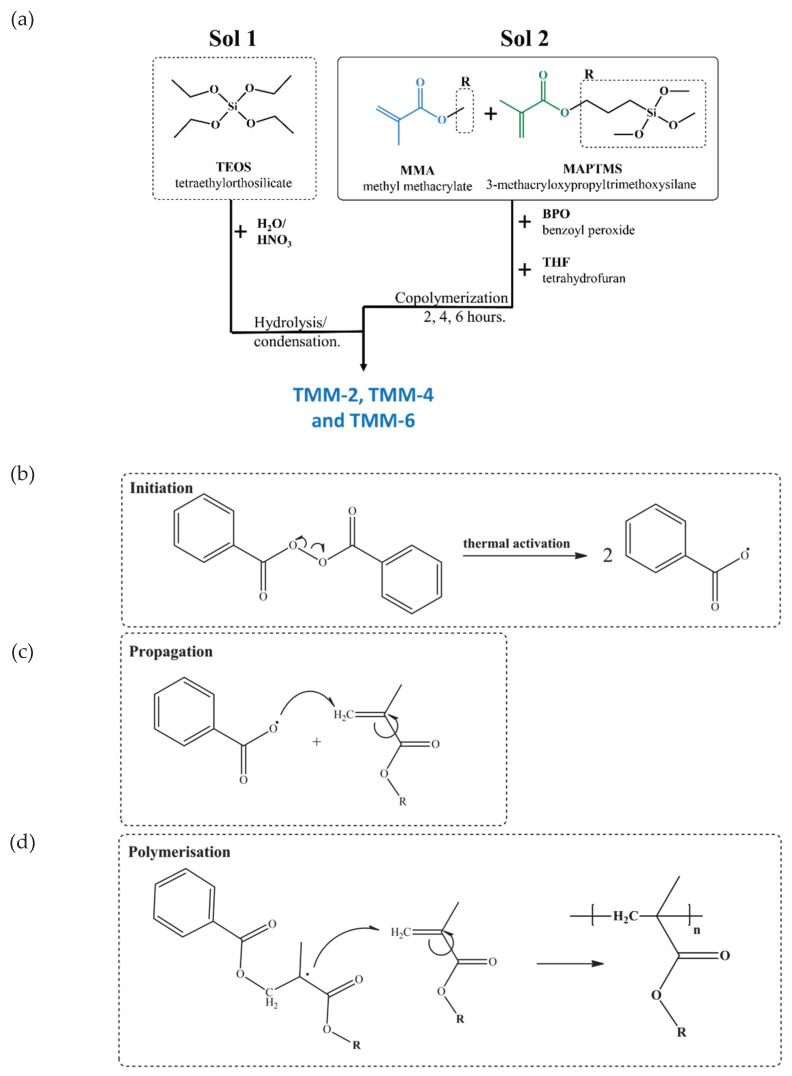
(**a**) Flow chart of the hybrid sol-gel preparation; (**b**) thermal initiation of benzoyl peroxide BPO; (**c**) propagation of the prepared radical; and (**d**) copolymerization of acrylate groups. R is the remainder of the MMA and MAPTMS molecules, marked by the dashed square in Figure 1a.

Organic–inorganic (hybrid) sol-gel solutions were synthesised by combining two separately prepared solutions: hydrolysed/condensed TEOS (silica sol, Sol 1) and copolymerised MMA/MAPTMS sol in THF (Sol 2) (Figure 1a).

The molar ratio of the reagents used was: TEOS:EtOH:H_2_O:MMA:MAPTMS:BPO:THF = 2:5.3:10.5:8:1:0.08:53, which was a similar composition to reported as optimal in [27,29,37]. Water acidified with nitric acid (H_2_O/H^+^) had a pH = 1. Sol 1 was prepared in a separate, closed flask during dropwise addition of ethanol and H_2_O/H^+^ into TEOS followed by mixing for 30 min at ambient temperature. The radical copolymerization process between MMA and MAPTMS in the presence of BPO and THF was performed in a 50 mL closed batch reactor using an EasyMax laboratory reactor system (EasyMax 102 workstation) under controlled air atmosphere at 70 °C; the reactor jacket was heated with a heating rate of 10 °C/min and kept constant at 70 ± 0.1 °C for various copolymerization times (2, 4 and 6 h). The reactor jacket was cooled to room temperature at a cooling rate of 10 °C/min. The solution was mixed vigorously using the cross-shaped Teflon magnetic stirring bar. In the last step, Sol 1 was added dropwise to Sol 2 during vigorous mixing (Figure 1a). The prepared solution was further mixed for 1 h at ambient temperature to obtain the final sol-gel solution.

Organic–inorganic (hybrid) sol-gel/coatings synthesised from TEOS, MMA and MAPTMS are denoted as TMM-*x*, where *x* reflects 2, 4 and 6 h of copolymerization between MMA and MAPTMS (TMM-2, TMM-4 and TMM-6).

### 2.3. Sample Preparation

AA7075-T6 sheets, distributed by Kaiser Aluminum Corporation, California, USA, were cut into smaller pieces with a size of 20 mm × 40 mm × 0.5 mm. The certified composition of the alloy is 89.41 Al, 5.81 Zn, 2.55 Mg, 1.67 Cu, 0.21 Fe, 0.19 Cr, 0.08 Si and 0.08 wt.% other admixtures. The surface was ground mechanically using the grinding/polishing machine (LaboSystem, LaboPol-20, Struers, Cleveland, OH, USA) to give a smooth surface without visible tracks of grinding. The surface was ground using, in sequence, 1200, 2400 and 4000 grit waterproof silicon carbide grinding papers (Struers, Cleveland, OH, USA). The sample was then cleaned ultrasonically in ethanol for 5 min (Elma ultrasonic cleaner Elmasonic S10H) to remove all the grinding residues, rinsed with ethanol and, finally, dried with a stream of compressed nitrogen.

The coatings were applied to a substrate using a one-step dip-coater process. The samples were attached to the holder of the dip-coater and dipped into the solution, which was placed in a glass beaker. The dip-in and pull-out rates were adjusted to 14 mm/min. The sample was dipped in the solution for 3 s. The coating was deposited once (i.e., one layer). After deposition, the coating samples were cured thermally in a preheated oven at 60 °C for 30 min and, later, cured at 166 °C for 1 h to give a final coating ready for further characterisation.

### 2.4. Characterisation of the Synthesis

#### 2.4.1. Fourier Transform Infrared Spectroscopy

Chemical changes during the sol-gel synthesis in a closed 50 mL batch reactor (Sol 1, Sol 2, Sol 1 + Sol 2 and final TMM sols) were analysed by a real-time Fourier transform infrared (FTIR) spectrometer in a range from 2400 to 650 cm^−1^. The spectra were recorded with a ReactIR™ 45 instrument using a universal attenuated total reflectance (ATR) sampling accessory with a resolution of 4 cm^−1^, averaging 128 scans. An EasyMax 102 controller was used to control reaction conditions during the reaction. The instruments were controlled by iControl EasyMax 4.2 and iC IR 4.2 software. Spectra are given in absorbance units (A.U.).

#### 2.4.2. Dynamic Light Scattering

Dynamic light scattering (DLS) was carried out for evaluation of the hydrolysis/condensation of TEOS, the copolymerization process of MMA/MAPTMS, and the final TMM sols, using a standard photon correlation setup with a frequency-doubled diode-pumped Nd–YAG laser and an ALV-6010/160 correlator, to obtain the autocorrelation function of the scattered light intensity. The measurements were performed at room temperature and at a scattering angle of 90°. In most cases, the decay of the autocorrelation function consisted of a distribution of relaxation processes, which corresponds to the diffusion of the constituents. The data were analysed using the CONTIN analysis [57], which is part of the ALV-Correlator Software V.3.0. This analysis gives the distribution function of the relaxation times, which are, in dilute samples, proportional to the size of the constituents. In more concentrated samples or those undergoing polymerisation or gelation, diffusion of the constituents is hindered and/or affected by the change of effective viscosity; the size distribution of the constituents cannot be determined reliably. For that reason, the DLS results are represented by the distribution function of the relaxation times.

#### 2.4.3. Gel Permeation Chromatography

The average molecular weight (M_w_), the number average molecular weight (M_n_) and the polydispersity index (PDI = M_w_/M_n_) for the copolymerization process of MMA/MAPTMS and the final TMM sols were determined by gel permeation chromatography (GPC), using a Waters 2690 instrument (Separations Module) equipped with an isocratic pump, an auto-sampler injector, a column oven and an RI detector Waters 410 (Differential refractometer). A set of three Waters Styragel columns (Styragel HR 5E, Styragel HR 4E and Styragel HR 0.5) (300 × 4.6 mm, 5 μm) was connected, in front, with a Waters Styragel guard column (30 × 4.6 mm). Samples were dissolved in anhydrous THF, and then injected into the chromatograph. The column oven was maintained at 40 °C. THF was used as the eluent at a flow rate of 0.2 mL min^−1^. The GPC system was calibrated with a narrow molar mass of polystyrene (PS) standard by PL (Polymer Laboratories, Amherst, ZDA). All molar mass values obtained are, thus, relative to this standard. The data were processed by the Waters GPC program.

#### 2.4.4. Thermal Analysis

Coupled thermogravimetric Analysis/Mass Spectrometry measurements (TG-MS) were performed for TMM-4 on a Mettler Toledo TGA/DSC1 instrument connected to a Pfeiffer Vacuum ThermoStar mass spectrometer. About 3 mg of the sample were placed into a 150 µL platinum crucible and heated at a rate of 10 °C min^−1^ from 25 to 550 °C. During the measurement, the furnace was purged with air with a flow rate of 50 mL min^−1^. The empty crucible served as a reference. The blank curve was subtracted. Evolved gases were introduced into the mass spectrometer via the 75-cm long heated capillary.

The coating was also analysed with High-Pressure Differential Scanning Calorimeter (HP DSC). Measurements were carried out on a Mettler Toledo DSC 827^e^ instrument in 40 μL standard aluminium pans in the static air atmosphere. The temperature ranged from 30 to 250 °C, the heating rate was 10 °C min^−1^ and the initial pressure 50 bars. An empty pan served as a reference. Weights of dry samples were between 1.5 and 2.5 mg.

#### 2.4.5. Physico-Chemical Characterisation

The density (*γ*) of sols was determined at 25 °C using a densimeter DMA 5000 (Anton Paar Ltd. Paar Scientific, Ltd., London, United Kingdom). The instrument was calibrated using ultra-pure water. Another important parameter was the viscosity of the fluid, which is the internal resistance to flow. It is usually given as kinematic viscosity (how fast the fluid is moving) when the fluid is subjected to deformation) and dynamic viscosity (the force required to maintain the fluid flow at a particular rate). The viscosities of the TMM sols were measured by adding 2 mL of sol to an Ubbelohde capillary viscometer Type-Nr. 517 20 that was then dipped into the isolated thermostated oil bath at 25 °C. The bath temperature was controlled by a Lauda Eco Silver thermostat. The measurements were performed after stabilisation for 10 min. The liquid was pumped into the capillary using an AVS 370 pumping machine. The lowering of the level of fluid was measured with two photocells, where the time between two different level points was determined using AVS 370 software–Version 3.79.Bn. The kinematic viscosity, *ν*, given in Stokes (St = mm^2^/s), of the liquids was calculated using the instrument constant in Equation 1:

*ν* = K × *t* (1)

where *t* is the flow time in seconds, and K the instrument constant valid for survey of the meniscus passage. K = 0.1111 mm^2^/s^2^, is the instrument constant valid for liquids with a surface tension of 20 to 30 mN/m and an acceleration of the fall of 9.8105 m/s^2^. The measurements were repeated five times. The results are given as average values ± standard deviations. The dynamic viscosity, given in mPa s, was calculated as the product of kinetic viscosity and fluid density, *γ*, according to Equation (2):

*μ* = *ν* × *γ* (2)

### 2.5. Coating Characterisation

#### 2.5.1. Morphology

The morphology of the TMM coatings deposited on AA7075-T6 was analysed using a field emission scanning electron microscope (FESEM) FEI HeliosNanolab 650 coupled to an energy dispersive X-ray spectrometer (EDS) Oxford Instruments X-max SDD (50 mm^2^) detector using Aztec software. The FESEM micrographs were recorded using the secondary electron imaging (SEI) mode at 2 kV energy. The spots were analysed by EDS at 10 kV beam energy. Prior to analysis, the coating was scribed using the diamond blade; the coating thickness was determined at the scribe. The samples were sputter-coated with a layer of carbon, using a sputter coater BAL-TEC SCD 005.

#### 2.5.2. Corrosion Performance

Corrosion performance was tested in dilute Harrison’s solution [58] (3.5 g/L (NH_4_)_2_SO_4_ + 0.5 g/L NaCl) using electrochemical impedance spectroscopy (EIS). Measurements were made in a three-electrode corrosion cell placed in a Faraday cage. An area of 1 cm^2^ of the sample was exposed to the corrosive solution. As the reference electrode, a silver/silver chloride (Ag/AgCl, sat. KCl, *E* = 0.197 V vs. saturated hydrogen electrode) was used. A carbon rod served as a counter electrode. Electrochemical experiments were carried out with an Autolab PGSTAT302N (Metrohm Autolab, Utrecht, The Netherlands) potentiostat/galvanostat and controlled by Nova 1.11 software. EIS measurements were acquired in the frequency range from 100 kHz to 10 mHz with a 10 mV amplitude signal. Prior to measurement, the open circuit potential (OCP) was measured for 10 min. The EIS measurements were performed at the OCP, the first after 1-h immersion and then after regular intervals up to 4 months.

## 3. Results

### 3.1. Synthesis of the Sol-Gel Solution

Sols were synthesised following the three-step process: (i) hydrolysis/condensation reactions of alkoxide/silanol groups in TEOS (Sol 1); (ii) copolymerization between acrylate groups of MMA and MAPTMS in the presence of BPO (Sol 2); and (iii) hydrolysis/condensation reactions between alkoxide/silanol groups of MAPTMS and TEOS to obtain the final sol (TMM), ready for deposition on aluminium substrate. A flow chart of the individual steps of the synthesis and the thermal initiation of BPO is presented schematically in Figure 1a. Chemical changes during these three steps were followed by real-time FTIR, DLS and GPC techniques.

#### 3.1.1. Hydrolysis and Condensation of TEOS Studied by FTIR and DLS

**Figure 2:**
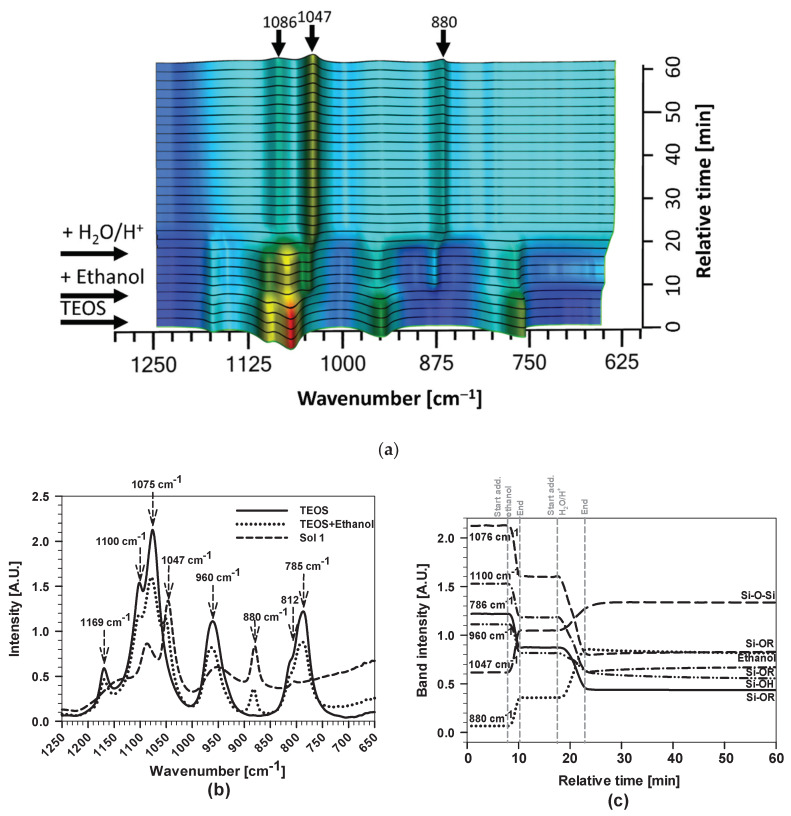
(**a**) Real-time 3D FTIR spectra and (**b**) 2D FTIR spectra after the addition of ethanol and after various times of hydrolysis/condensation process of TEOS following the addition of H_2_O/H^+^ to obtain Sol 1; and (**c**) band intensities of ν(Si–OR), ν(Si–OH) and ν(Si–O–Si) of the FTIR spectra during the hydrolysis/condensation process. The grey vertical dashed lines mark the start and end of the addition of ethanol and H_2_O/H^+^, respectively. Relative time represents the duration of the reaction step from starting to the end point.

The hydrolysis of an ethanol solution of TEOS, which proceeded under acid conditions (H_2_O/H^+^, pH = 1), led to the formation of Sol 1. FTIR spectra were recorded using real-time FTIR spectrometer, for TEOS alone, after the addition of ethanol, and 30 min after the addition of H_2_O/H^+^ (Sol 1) (Figure 2).

The most intense bands in the spectrum of TEOS, located at 785, 960, 1075, 1100 and 1169 cm^−1^, are related to C–H, Si–O and O–C bands (Figure 2a,b), as discussed in earlier publications [32,37,59]. After addition of ethanol to the TEOS solution, the spectrum contains the characteristic bands of ethanol, mainly seen as three strong bands at 880, 1047 and 1088 cm^−1^. The spectrum was the sum of bands characteristic for both components, although there were differences in the band intensities due to the impact of dilution. No change in positions of bands was observed during mixing.

After dropwise addition of H_2_O/H^+^ to the solution, hydrolysis of ethoxy groups (CH_3_CH_2_O–Si(OCH_2_CH_3_)_3_) started and silanol groups (HO–Si(OCH_2_CH_3_)_3_) were formed. At the same time, the polycondensation between two silanol groups occurred and siloxane ((CH_3_CH_2_O)_3_Si–O–Si(OCH_2_CH_3_))_3_) species were formed, leading to the formation of Sol 1 (Figure 2a,b). The intensity of bands generally decreased, especially that at 1078 cm^−1^ corresponding to Si–O–R groups, and to other TEOS bands in the range between 750 and 850 cm^−1^. Siloxane (Si–O–Si) related bands appeared in the range between 850 and 1050 cm^−1^ and became the main bands of the spectrum. No significant differences were observed at wavenumbers higher than 1250 cm^−1^ (the spectra are not shown).

The process can be further analysed by comparing the characteristic band intensities as a function of time (Figure 2a,c). The first change in the bands’ intensities is due to dilution following the addition of ethanol and the second to the addition of H_2_O^+^/H^+^. The increase of the bands related to the Si–O–Si network (1047 and 1086 cm^−1^) occurred at the expense of the Si–O–R bands of TEOS at 1100, 1076 and 786 cm^−1^ and the band of Si–OH (at 960 cm^−1^) (Figure 2c). The latter is assigned to transverse optic modes, corresponding to different structural units with smaller siloxane, and larger Si–O–Si angles and Si–O bond lengths [32,59]. At the same time, the band at 880 cm^−1^, corresponding to ethanol, increased in intensity due to its formation as a side product of the hydrolytic condensation of TEOS. The existence of residual Si–O–R (at 1076 cm^−1^) and of Si–OH (at 960 cm^−1^) groups, even after 30 min of mixing, indicates that, although the sol was stabilised, the hydrolysis/condensation reactions were not complete at the end of the first stage of the synthesis process. During mixing, bands at 1047 and 1086 cm^−1^, related to the linear Si–O–Si chain, still increased (Figure 2a,c), whereas the broad band at 960 cm^−1^, related to Si–OH group, slowly decreased.

To sum up, the hydrolysis/condensation reactions of TEOS proceeded rapidly under the reaction conditions used. The process was stabilised after 30 min of mixing, but was still not complete (i.e., TEOS was not fully hydrolysed/condensed).

**Figure 3:**
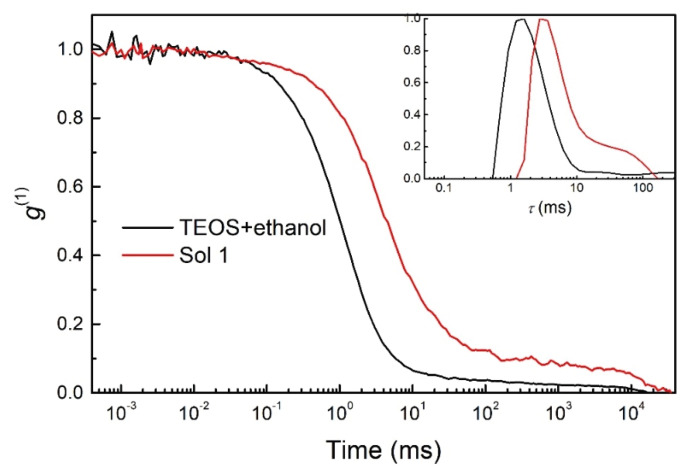
Autocorrelation functions (*g*^(1)^) as a function of delay time obtained by DLS showing the diffusion of species for TEOS+ethanol and TEOS+ethanol+H_2_O/H^+^ (Sol 1) after 30 min of hydrolysis/condensation process. Inset: The distribution of relaxation times (*τ*) showing significantly larger cluster size in Sol 1.

The hydrolysis/condensation process prior to hydrolysis (TEOS+ethanol) and after 30 min of mixing TEOS+ethanol+H_2_O/H^+^ to obtain Sol 1 was additionally characterised by Dynamic Light Scattering (DLS) measurements (Figure 3).

According to the literature [60,61,62], species in the nanometre range are expected when the synthesis is performed in ethanol solution in the presence of H_2_O/HNO_3_. Their size and distribution are affected mainly by acid, water and alcohol ratios [60]. The autocorrelation functions (*g*^1^) for this system showed one distinct dynamic process or, more specifically, the changes in the diffusion of species, which are shifted to higher values by almost one order of magnitude. The clusters of Si–O–Si formed confirm the presence of hydrolysis/condensation reactions, which is consistent with the analysed real-time FTIR spectra (Figure 2). Moreover, the data confirm the formation of species with hydrodynamic radii in the nanometre range. The results presented in the inset show the distribution of relaxation times (τ) with significant larger cluster size in Sol 1. The narrow peak indicates presence of distributions of formed clusters with similar size. However, due to the high concentration in ethanol, the size of the species cannot be estimated more precisely.

#### 3.1.2. The Copolymerization Process between MMA and MAPTMS, Studied by FTIR, DLS and GPC

In the copolymerization step of Sol 2, solutions of the appropriate amounts of MMA, MAPTMS, BPO and THF were mixed in the closed batch reactor (Figure 1a). The reactions were carried out at 70 °C under reflux to initiate the decomposition of BPO to benzoyl radicals (Figure 1b). Once free radicals were formed, the polymerisation propagated, with free-radicals attacking the vinyl (double) bond (C=C) of methacrylate monomers of MMA and MAPTMS until the polymerisation was terminated by another free radical (Figure 1c). Finally, the non-terminated, polymeric-free radical served as a macroinitiator that reacted with another molecule, resulting in copolymers with polyacrylate segments to result in final copolymerization of MMA and MAPTMS (Figure 1d).

**Figure 4:**
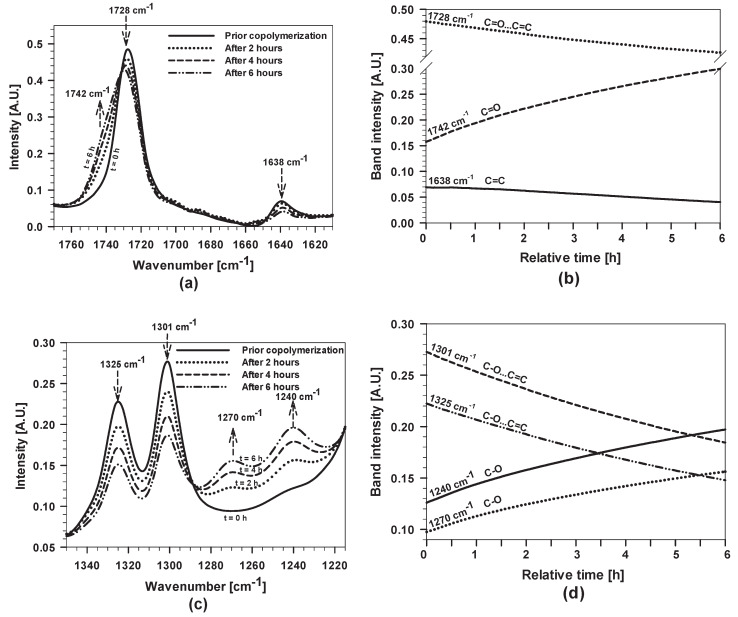
(**a**,**c**) FTIR spectra of the copolymerization process of MMA/MAPTMS in the presence of THF and BPO before and after various copolymerization times ranging from 2 to 6 h; and (**b**,**d**) the kinetic profiles of the intensity of ν(C=C), ν(C=O) and ν(C–O) bands during the copolymerization of acrylates (the first stage of synthesis) for 6 h. Relative time represents the duration of copolymerization from starting to end point at 70 °C.

**Figure A1:**
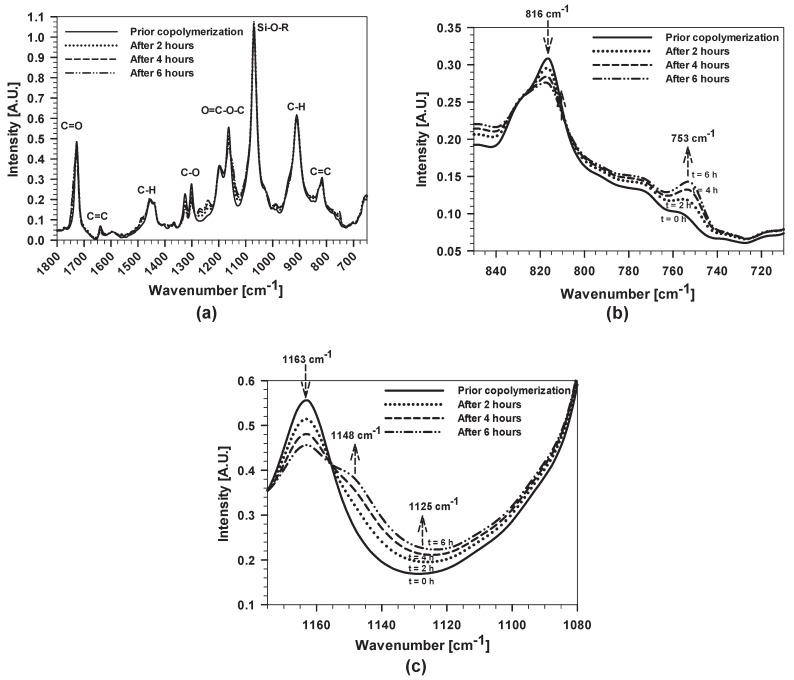
(**a**) Real-time FTIR spectra of the copolymerization process of MMA/MAPTMS in the presence of THF and BPO during the process of acrylate copolymerization (first stage of synthesis) and during the first 6 h; (**b**) the inset of ν(C=C) bands; and (**c**) ν(C–O–C) bands.

The copolymerization was followed by the characteristic bands in real-time FTIR spectra before and after various times of copolymerization ranging from 2 to 6 h. The FTIR spectra recorded in the broad range are shown in the Appendix A (Figure A1) and detailed spectra are presented in Figure 4.

The spectrum prior to copolymerization shows the presence of characteristic bands for MMA, MAPTMS and THF [37]. The most crucial changes accompanying increasing copolymerization time occurred in the characteristic regions of vinyl C=C bands (at 1638 and 816 cm^−1^) and the CH_2_ band (at 1453 and 753 cm^−1^). Additionally, the C=O stretching band at 1728 cm^−1^, the antisymmetric stretching bands of C–O at 1325 and 1301 cm^−1^ and O=C–O–C at 1163 cm^−1^ changed in intensity and peak position (Figure A1). The intense band at 1170 cm^−1^ is related to Si–O–R.

To quantify the changes occurring during the copolymerization process, the most characteristic bands, i.e., C=O, C=C and C−O and CH_2_, were followed as a function of copolymerization time by real-time FTIR (Figure 4 and Figure A1). The intensity of the C=C band at 1638 cm^−1^ decreased with increasing copolymerization time (Figure 4a). At the same time, changes were observed for the C=O band located at 1728 cm^−1^, assigned to the C=O stretching vibrations that are conjugated to C=C double bonds. During acrylate copolymerization, the shift of the C=O band to higher wavenumbers took place (to 1742 cm^−1^). The copolymerization process was reflected at lower wavenumbers as well, between 720 and 840 cm^−1^ (Figure A1b). The band at 816 cm^−1^, related to C=C, decreased, whereas the band at 753 cm^−1^ increased due to the formation of CH_2_ bonds.

Figure 4b shows the intensities of the characteristic bands during the copolymerization for 6 h presented in Figure 4a. The rate of copolymerization is time-dependent. During the first 2 h, a rapid increase in the intensity of the C=O shoulder at 1742 cm^−1^ was noted. At longer copolymerization times, the rate slowed down due to the formation of larger copolymerised species, which probably sterically hindered the copolymerization process. After 6 h, the active radical polymerisation process was still not complete, indicating that the copolymerization process stopped at a certain point. The process was slowed down after cooling the reactor to room temperature, but the process was not complete, since benzoyl radicals were still present in the solution.

Changes in the copolymerised structure also affected the position of the C–O band between 1200 and 1350 cm^−1^ (Figure 4c–d). The band’s intensities at 1325 and 1301 cm^−1^ decreased; in contrast, those at 1270 and 1240 cm^−1^ increased. The changes in the intensity of these bands were related to the copolymerization process of acrylate groups that also affected the behaviour of other groups and their interactions in the sol. Similar behaviour was also noted for the C–O–C band at 1163 cm^−1^, whose intensity decreased and those of bands at 1148 and 1125 cm^−1^ increased (Figure A1c).

To sum up, the intensity of the bands related to acrylate groups of initial MMA and MAPTMS monomers were reduced during the copolymerization process (1728, 1638, 1325, 1301, 1163 and 816 cm^−1^), whereas those of the characteristic bands assigned to copolymerised acrylate increased (1742, 1270, 1240, 1148, 1125 and 753 cm^−1^). It is important to point out that the copolymerization was not complete, but stopped at a certain level.

**Figure 5:**
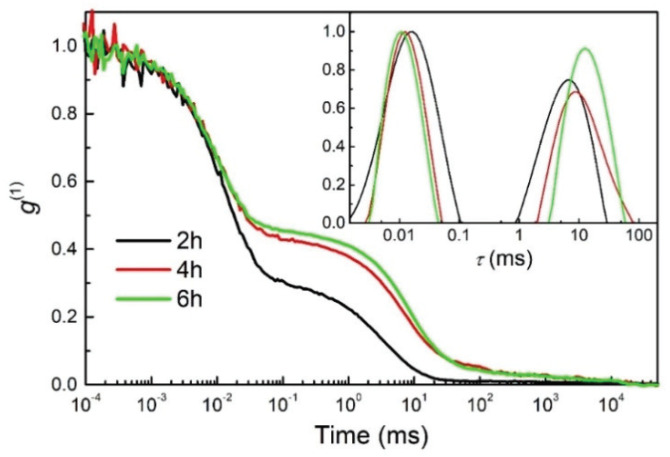
Autocorrelation functions (g^(1)^) as a function of delay time, measured by DLS, showing the copolymerization process of MMA/MAPTMS after various times: 2, 4, and 6 h. Inset: The distribution of relaxation times (τ) showing growth and consequent slowing of the diffusion of the clusters.

The copolymerization process was additionally characterised by measurements of DLS (Figure 5).

The autocorrelation functions showed the two dynamic processes characteristic of a polymerisation process. The rapid one corresponds to the dynamics of the sol constituents which, in our case, have a hydrodynamic radius of around 1 nm. The radius was estimated using the CONTIN analysis, where the estimated viscosity of 0.002 Pa.s was used. The second process, which is about three orders of magnitude slower than the fast one, is usually attributed to the dynamics of the larger clusters.

The data confirm the copolymerization process of the initial monomers of MMA and MAPTMS and the formation of larger polymer chain structures at increasing copolymerization time. After 2 h of copolymerization, aggregation started with the formation of shorter chains, observed in the relaxation time distribution function as a broad peak corresponding to the fast relaxation process. The slow relaxation process grew and slowed down with the time of copolymerization and, after 4 and 6 h, a high degree of copolymerization of acrylate groups and the formation of a long-chain network was observed (Figure 5). These results correlate with those obtained by real-time FTIR, where the copolymerization process was observed as a function of time to be higher at the beginning (first 2 h) and lower after a long copolymerization time (after 4 and 6 h) (Figure 4).

**Figure 6:**
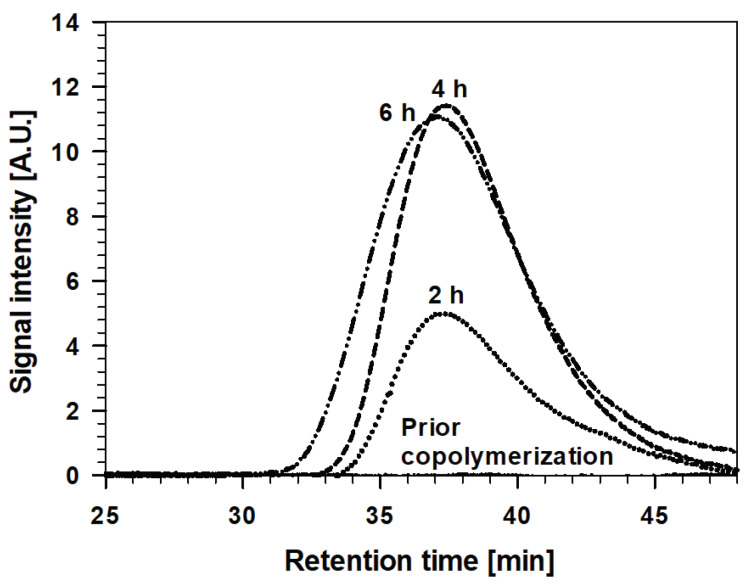
Gel permeation chromatograms of copolymerised MMA/MAPTMS prior to and after various copolymerization times: 2, 4 and 6 h.

The molecular weight distribution of MMA/MAPTMS was also characterised after various stages of the copolymerization process, using the GPC technique. The weight average molecular weight (M_w_), number average molecular weight (M_n_) and polydispersity index (PDI) were compared before and after the copolymerization (Table 1 and Figure 6).

The shape of curves, recorded before and after copolymerization as a function of retention time, indicates that copolymerised material was formed, and M_w_ of the TMM-2, TMM-4 and TMM-6 increase with copolymerization time. However, those recorded after 2 and 4 h were closely similar (M_w_ = ~82 kg/mol) but, after 6 h, increased substantially, especially in M_w_ (M_w_ = 147.6 kg/mol) and PDI, despite the M_n_ remaining similar for all copolymerised solutions. Such behaviour can be explained by the formation of macromolecules among the previously formed molecules, which correlates with the polymerisation process of the initial monomers (as also observed in the FTIR and DLS results, Figure 4 and Figure 5).

#### 3.1.3. The Mixing of Copolymerised Acrylates and Hydrolysed/Condensed TEOS Studied by FTIR, DLS and GPC

**Figure 7:**
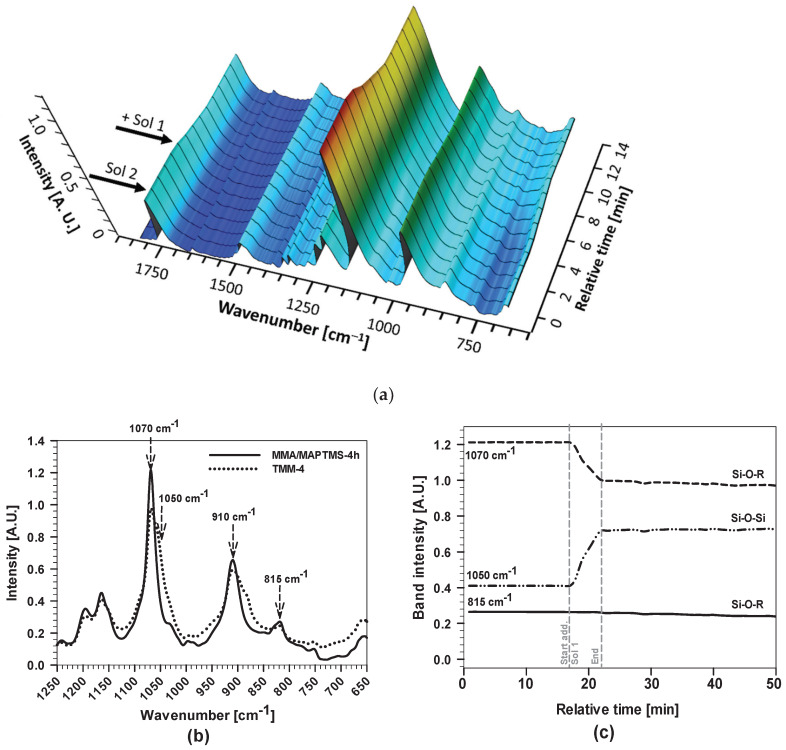
(**a**) Real-time 3D FTIR spectra and (**b**) 2D spectra of hydrolysation/condensation of MMA+MAPTMS (Sol 2) after addition of hydrolysed TEOS (Sol 1) to produce the final sol; and (**c**) the band intensities of ν(Si–O–R) and ν(Si–O–Si) bands of the FTIR spectra during the hydrolysis process to yield the final sol. The grey vertical dashed lines mark the start and end of the addition of Sol 2 to Sol 1. The relative time represents the duration of hydrolysis/condensation reactions (before, during and after addition).

The FTIR spectra were recorded during dropwise addition of hydrolysed/condensed TEOS (Sol 1) to copolymerised acrylates (Sol 2) to produce the final sols (Figure 1a). The mechanism of formation of TMM-solution was characterised using FTIR spectra for TMM-4 sol, given as a representative system (Figure 7a).

The intensities of some characteristic bands decreased because of dilution after the addition of Sol 1 to Sol 2. The main difference can be seen in the range between 650 and 1300 cm^−1^ (Figure 7a,b). This region represents the contribution of different vibrational bands: the Si–O–Si stretching modes from the oligomeric precursor as well as the C–O associated with the methoxy silyl functions. After the addition of Sol 1, the intensity of the characteristic bands for MAPTMS at 1070, 910 and 815 cm^−1^ decreased. The evidence for progressive condensation reactions is reflected by the increase in absorbance of the bands assigned to siloxane bonds, seen as a shoulder at the lower frequencies at 1050 cm^−1^ assigned to Si–O–Si bands.

The intensities of characteristic bands as a function of time are presented in Figure 7c. The reaction took place rapidly, seen as a decrease of band intensity at 1070 and 815 cm^−1^ as well as the increase of the intensity of the characteristic band at 1050 cm^−1^. However, the intensities of the bands at 1070 and 815 cm^−1^ slowly decreased further due to the hydrolysis process of MAPTMS, and the band at 1050 cm^−1^ increased due to the formation of Si–O–Si bands. After 1 h, the intensities of the bands did not change with time, meaning that the stable TMM sol-gel was formed.

**Figure A2:**
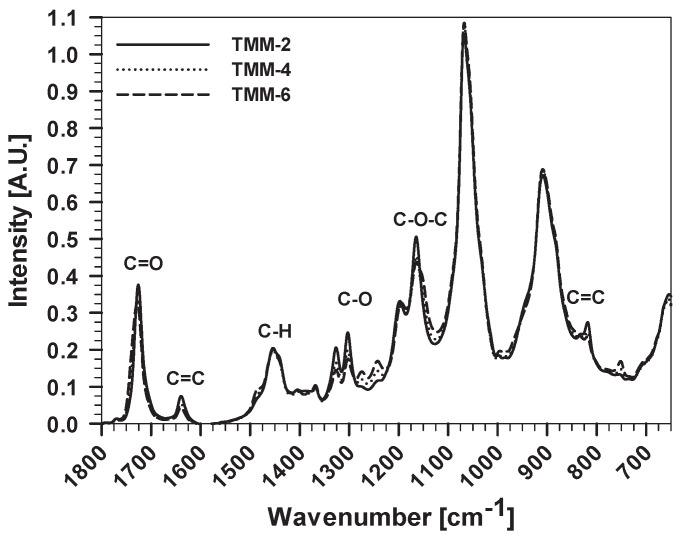
Real-time FTIR spectra of the TMM-2, TMM-4 and TMM-6.

In general, all the TMM-*x* sols have similar FTIR spectra (Figure A2). The main differences were observed in the region characteristic of C=C bands and C=C…C=O interactions (bands at 1728, 1638, 1301, 1325, 1270 and 1240 cm^−1^), which are related to the copolymerization process. There were no significant differences in the region related to Si–O–Si bands related to hydrolysis/condensation reactions. Unfortunately, the presence of water, which could be evidenced by a distinctive band at 1640 cm^−1^_,_ cannot be determined quantitatively due to its overlapping with the C=C band (the spectra are not shown).

**Figure 8:**
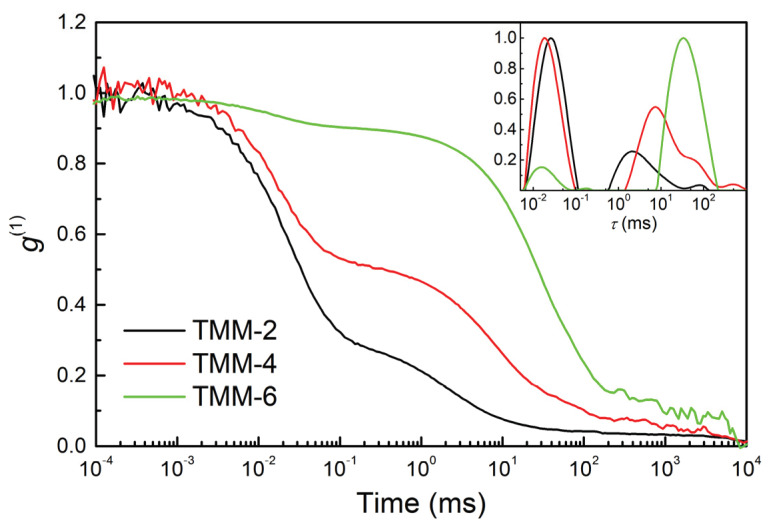
Autocorrelation functions (*g*^(1)^) as functions of delay time measured by DLS, showing the copolymerization process of TMM-2, TMM-4 and TMM-6. Inset: Distribution of relaxation times (*τ*) showing growth and consequent slowing of the diffusion of the clusters.

The TMM-2, TMM-4 and TMM-6 solutions were further characterised using DLS measurements (Figure 8).

The autocorrelation functions again showed two distinct dynamic processes. The fast one corresponds to the dynamics of the sol constituents, while the second process is attributed to the dynamics of the copolymerised clusters. From 2 to 6 h, their diffusion slowed down by almost two orders of magnitude, which is a consequence of the clusters’ growth (Si–O–Si network) and especially the formation of the polymer network between acrylates.

**Figure 9:**
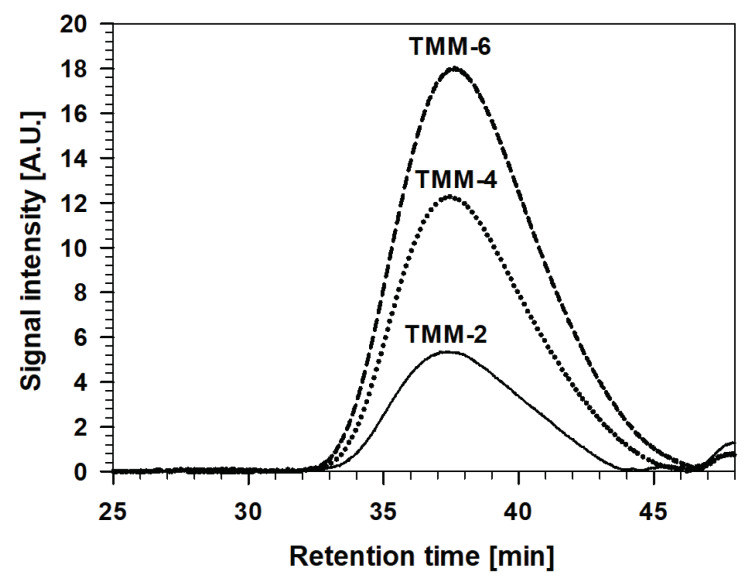
Gel permeation chromatograms of TMM-2, TMM-4 and TMM-6.

Gel permeation chromatograms for the TMM-2, TMM-4 and TMM-6 solutions are presented in Figure 9.

Comparison of Sol 2 and the final TMM sols reflects the similar retention times for the two solutions, seen as slightly smaller M_w_ and PDI and greater M_n_ (Table 1). Such behaviour can be explained by the addition of Sol 1 (containing ethanol) to Sol 2. However, there are some differences in the properties of TMM-*x* sols. The levels of M_W_ and PDI increased slightly from TMM-2 to TMM-6, while that of M_n_ decreased in the opposite direction. It appears that the addition of Sol 1 to Sol 2 led mainly to the formation of small molecules due to hydrolysis/condensation reactions; on the other hand, the copolymerization remained at the same level.

### 3.2. Solution Characterisation

#### 3.2.1. Sol Density and Viscosity

The prepared sols were stable for a few hours, clear and transparent. Their density differs depending on the copolymerization time (Table 2).

The TMM-2 sol-gel solution had a density of 0.924563 g/mL. The density of TMM-4 decreased slightly due to the longer copolymerization time and the controlled hydrolysis/condensation reactions, where Si–O–Si constitutes a spacer between the copolymerised species. However, with an increase in copolymerization time (TMM-6), the density again increased due to the formation of larger copolymerised species in the solution (higher degree of copolymerization, Table 1) which probably reduced the formation of Si–O–Si spacers. The results obtained correlate with those obtained with DLS and GPC.

The second important parameters are the kinematic and dynamic viscosities of a fluid, which describe the resistance to gradual deformation by shear stress or tensile stress. These parameters are important for the application of a sol in the form of a coating. TMM-2 has the lowest kinematic (2.68 St) and dynamic (2.48 mPa s) viscosities compared to those of TMM-4 and TMM-6. This behaviour indicates that a higher degree of cross-linking increases the kinematic as well as the dynamic viscosity of the sol. The viscosity is also related to the degree of hydrolysis of the alkoxy groups and to the condensation reactions between silanol groups, but this effect cannot be evaluated directly from these measurements.

#### 3.2.2. Thermal Analysis

**Figure 10:**
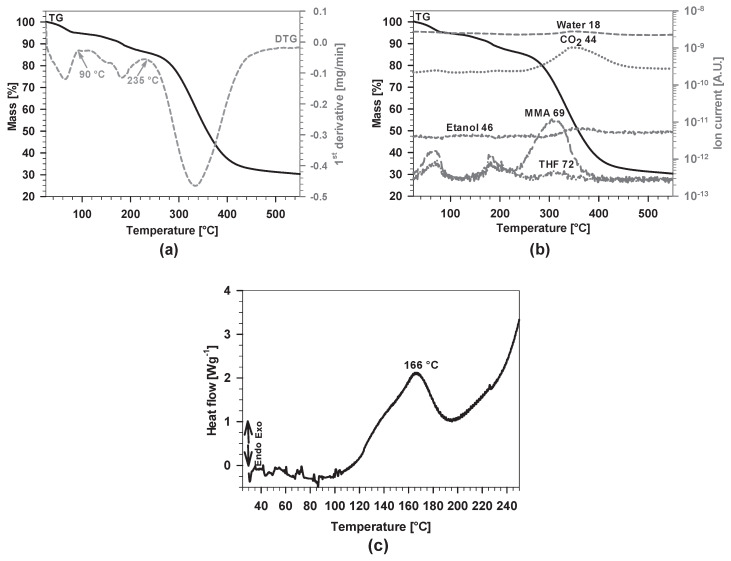
(**a**) TG and DTG curves of the studied samples TMM-4 under air atmosphere with a flow rate of 50 mL/min and a heating rate of 10 °C/min; (**b**) MS analyses of evaporated compounds with the TG curve added as a reference; and (**c**) HP DSC curve of TMM-4 dried gel under static air atmosphere. The initial pressure in the DSC cell was 50 bars.

The thermal stability of the TMM hybrid sol-gel solutions prepared at various copolymerization times and the course of their thermal decomposition were studied using coupled thermoanalytical methods, thermogravimetry–mass spectrometry (TG-MS). Figure 10a–c shows the TG-DTG, TG-MS and HP DSC curves for TMM-4, which was used as representative due to the same molar composition as that for the other two sols, which showed similar behaviour.

From room temperature up to 550 °C, three successive steps took place that partially overlap. From the DTG curve (Figure 10a), one can observe the temperature range of the individual steps. During the first step, from room temperature up to 90 °C, the excess free MMA (69 *m/z*) and solvent THF (72 *m/z*) evaporated from the sample (Figure 10b); mass loss was 5.0%. In this range, signal *m/z* = 44, typical for CO_2_, also appeared, most probably due to strong electron ionisation in the orifice of the mass spectrometer, which caused fragmentation of larger molecules. The intensity of the signal of water molecules (18 *m/z*) was low due to the high background in the mass spectrometer.

Mass loss in the second step, ranging from 90 to 235 °C, was 10.0%. Signals detected by the mass spectrometer showed clearly the evolution of MMA and THF while, under closer inspection, the evolution of ethanol (46 *m/z*) indicated condensation reactions between Si–OH and Si–OCH_2_CH_3_ in the formed network.

The temperature range of the polycondensation reaction was determined using high pressure DSC. Exothermic effects, occurring during curing reactions, are usually hidden at ambient pressure due to the endothermic evaporation of small gaseous species that takes place at the same time. The elevated pressure prevents evaporation of small molecules and, consequently, enables the detection of this type of reactions. In Figure 10c, a broad exothermic peak appears in a temperature range from 100 to 200 °C. It corresponds to the second step on the TG curve (Figure 10b). The temperature of the maximum at 166 °C was identified as the optimal curing temperature. This finding confirms the condensation process of silanol groups to form the final coating network.

The third step, ranging from 235 to around 450 °C, is attributed to the evaporation of the THF and MMA captured in the polymerised network. The random chain scission and combustion of organic compounds in the hybrid material, for which formation of CO_2_ is typical, also took place within this temperature range, with a mass loss of 55.6%. The temperature of the most intense evolution of THF and MMA was 310 °C, while for CO_2_ and water molecules the temperature was shifted to 345 °C, meaning that the above-mentioned reactions occurred successively. It should be noted that the duration of the curing reaction of the prepared hybrid sol-gel coating at 166 °C was 120 min. Supposedly, during this time, the majority of all THF and excess MMA within the formed network were evaporated.

### 3.3. Coating Characterisation

#### 3.3.1. Morphology, Composition and Thickness

**Figure 11:**
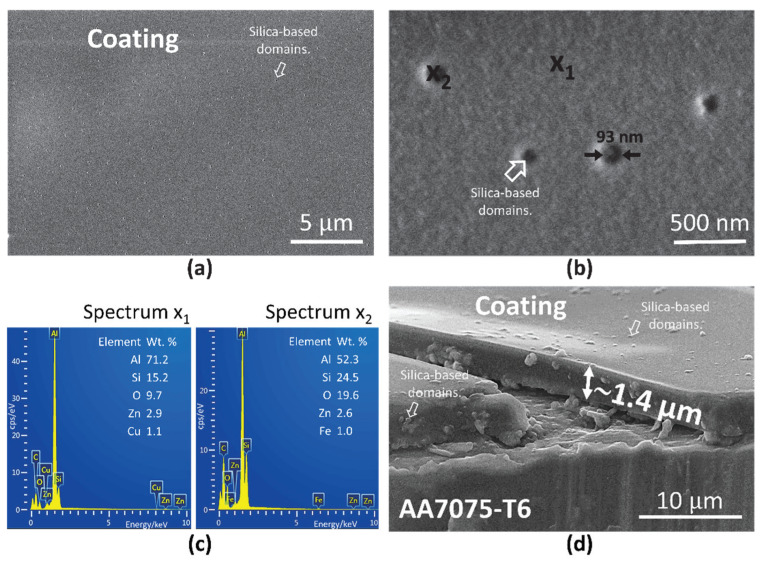
Top-view FESEM images of the TMM-4 coating deposited on AA7075-T6 (**a**) at low and (**b**) at high magnification. The arrows show the estimated size of silica-based domains. The enumerated spots (x_1_ and x_2_) denote positions, where the EDS analysis was performed. (**c**) Spectra x_1_ and x_2_ present compositions given in weight percentage (wt. %). (**d**) FESEM image along the artificially performed cross-section. The estimated coating thickness is around 1.4 μm. The arrows show the silica-based domains and their estimated size.

**Figure A3:**
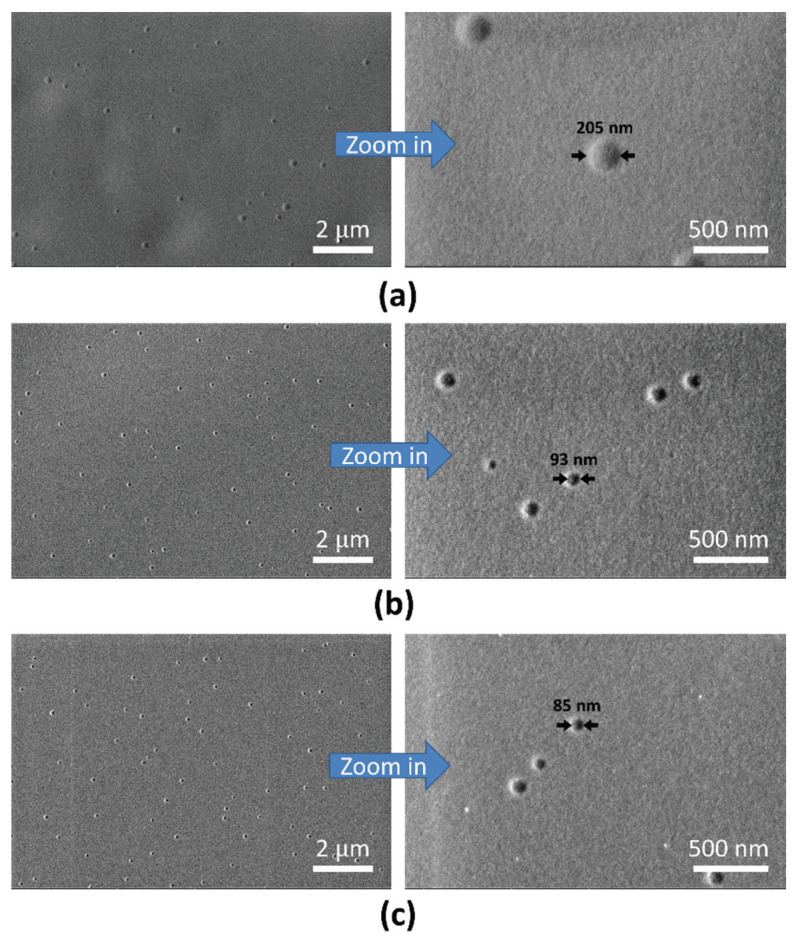
Top-view FESEM images of: (**a**) TMM-2; (**b**) TMM-4; and (**c**) TMM-6 coatings deposited on AA7075-T6 at low (**left**) or high (**right**) magnification. The arrows show the silica-based domains and their estimated size as formed in the coating.

The TMM-2, TMM-4 and TMM-6 coatings were analysed using FESEM/EDS (Figure A3 and Figure 11). The surface of the coating is very smooth, without any crack, flaking or visible pores, indicating that the coating covers the alloy surface evenly.

The coating contains small, randomly spread, nano-sized silicon-based domains (Figure A3 and 11a,b). The differences in the composition of matrix and silicon-based domains were checked with point EDS analysis at the coating surface (Figure 11b,c). At spot x_1_, i.e., at the coating bulk, the composition of the coating, given in weight percentage, refers to Si (15.2 wt. %) and O (9.7 wt. %) as major elements in the organosilane matrix of the hybrid sol-gel coating, which consists of MMA and MAPTMS. Since the thickness of the coating is in the micrometre range, the beam goes through the coating (due to performing the analysis at 10 kV) and reaches the substrate. Consequently, the peaks for elements originating from aluminium alloy 7075-T6 (aluminium and intermetallic particles in this alloy, i.e., Zn and Cu) are also present in the spectra as well. On the other hand, at spot x_2_, which is positioned at the nano-domain, the composition differs from spot x_1_ (Figure 11b,c). At the spot x_2_, a higher amount of Si and O was detected than at the spot x_1_, i.e., Si (24.5 wt. %) and O (19.6 wt. %). This is related to silica-rich domain (Si–O–Si) based on TEOS. The aluminium and elements of intermetallic particles are still present in the spectrum x_2_.

The amount and size of these domains were affected by the time taken for copolymerization. Their amount increased in the following order: TMM-2 < TMM-4 < TMM-6. However, their size decreased in the opposite direction, from TMM-2 (205 nm) to TMM-4 (93 nm) and TMM-6 (85 nm) (Figure 11a,b and Figure A3). This confirms that the amount and growth of these domains are correlated with the degree of copolymerised MMA/MAPTMS, which affects the incorporation of the inorganic phase (Sol 1) into the organic phase (Sol 2). The silica-domains are comparable with those observed in similar hybrid sol-gel coatings (with similar TEOS/ethanol ratio) [38]. The main difference is their size, which is much smaller in the TMM-*x* coating system.

The coatings were also analysed, together with the artificially made scribe, to evaluate the morphology of the coating. As a representative sample, the FESEM micrograph for the TMM-4 coating on AA7075-T6 is given in Figure 11d. It is important to note that the silica-rich domains are spread randomly throughout the coating. This confirms the fact that that these domains were formed during sol-gel synthesis and were evenly deposited while applying the coating to the surface.

The thicknesses of the coatings, evaluated by FESEM, are given in Table 2. They increased by more than three-fold, i.e., from 0.8 (TMM-2) to 2.6 μm (TMM-6). Greater thickness of the coating is related to the higher viscosity (increased by copolymerization time) of the sol-gel solution.

#### 3.3.2. Corrosion Performance

**Figure 12:**
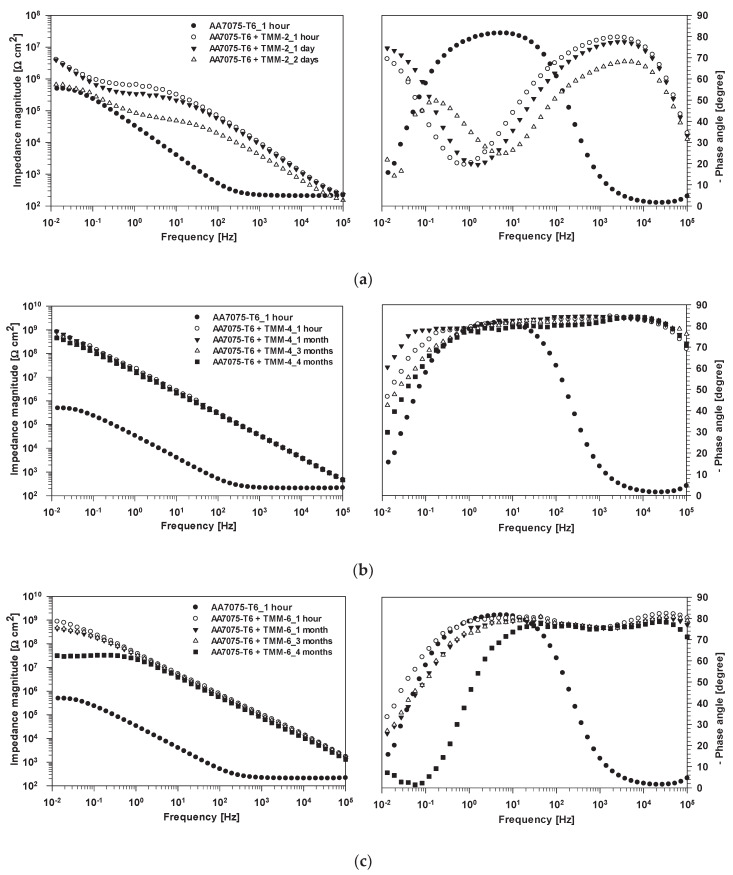
EIS spectra presented as Bode plots of (**left**) impedance magnitude and (**right**) phase angle for AA7075-T6 uncoated and coated with (**a**) TMM-2, (**b**) TMM-4 and (**c**) TMM-6 coatings recorded at different immersion times in dilute Harrison’s solution.

**Figure A4:**
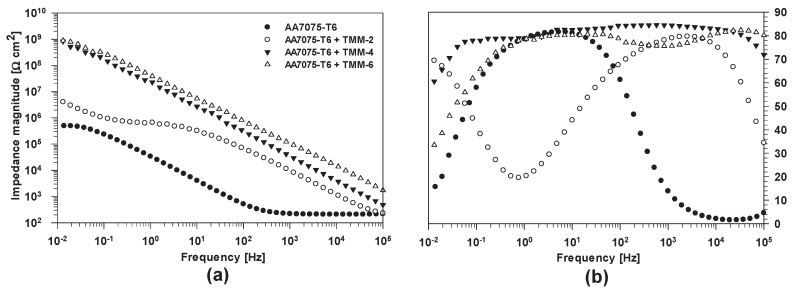
EIS results for uncoated AA7075-T6 and coated with TMM-2, TMM-4, and TMM-6 coatings immersed for 1 h in dilute Harrison’s solution presented as Bode plots of: (**a**) impedance magnitude; and (**b**) phase angle.

The corrosion performances of AA7075-T6 uncoated and coated with TMM-2, TMM-4 and TMM-6 were evaluated at regular intervals using electrochemical impedance spectroscopy. First, the Bode plots of impedance magnitude and phase angle recorded after 1 h of immersion in dilute Harrison’s solution were compared (Figure 12a–c and Figure A4).

Uncoated AA7075-T6 showed a small impedance value |Z_10 mHz_|= 200 kΩ cm^2^, and small phase angle at low frequencies, reflecting its relatively low corrosion resistance in dilute Harrison’s solution, despite this corrosion medium induced a less intensive attack than NaCl solutions [63]. The majority of the alloy surface was covered by an aluminium oxide layer, which provided resistance and capacitive contributions to the data obtained at the middle frequencies. The values of impedance and phase angle at frequencies between 1 and 0.01 Hz can be attributed to electrochemical (corrosion) processes near the metal surface [64]. The results confirm the conclusion that the corrosion activity on the aluminium surface is taking place already after 1 h of immersion, due to the reaction of Cl^−^ and SO_4_^2−^ ions with intermetallic particles [59,64,65], representing the initial stage of corrosion. To overcome this drawback, the alloy needs additional corrosion protection in the form of the coating.

All coated AA7075-T6 samples showed significantly higher impedance than the uncoated substrate, reflecting their enhanced corrosion resistance. According to the EIS theory [66,67], low frequency and medium-high frequency ranges reflect the barrier properties of the coating that protects the underlying substrate after the sample is exposed to an aggressive solution. Good barrier properties are an essential feature of highly effective anticorrosive coatings [27,39].

Several differences in EIS response were observed among TMM-*x* coatings. The TMM-2 coating exhibited an impedance magnitude value of |Z_10 mHz_|= 33 MΩ cm^2^, which is one order of magnitude higher than that of uncoated aluminium alloy. It is, however, substantially smaller than those of the other two coatings, TMM-4 and TMM-6. The values for the latter two are similar. The smaller impedance of TMM-2 is related to a shorter copolymerization time and thinner coating. The EIS data reflect the presence of some pores in the coating (Figure 12a) that allow the corrosion solution to penetrate the coating, as evidenced by the decrease in phase angle. On the other hand, coatings obtained after longer copolymerization times (MMA/MAPTMS copolymerised for 4 and 6 h) led to a sharp increase of the |Z_10 mHz_| above 1 GΩ cm^2^, which is more than three orders of magnitude greater than that of the uncoated aluminium. These coatings presented superior corrosive protection.

The values of phase angle were above −80° over a wide frequency range (over 5–6 decades in the mid- and high-frequency range), showing behaviour close to that known for a quasi-ideal capacitor. Values are related to the capability of the coating, which completely block the entrance of a corrosive medium (Figure 12b,c). The EIS data confirm that the time for copolymerization is a crucial step to achieve optimal copolymerization between MMA and MAPTMS (Sol 2).

The coatings were characterised as a function of immersion time to evaluate coating durability. TMM-2 coating lost its barrier properties after one day because a drop of the impedance magnitude was observed (Figure 12a), especially in the middle frequencies. After two days, a complete loss of barrier properties occurred due to progressive penetration of electrolyte through the coating and corrosion-induced defects (pits) in the coating. Thus, the lifespan of the coating is limited to only one day. Impedance values below 1 MΩ cm^2^ indicate weak barrier properties [39,68], because values are similar to that for the uncoated alloy. The low durability is related mainly to low thickness, low degree of copolymerization and the swelling effect of the organic phase in contact with corrosive media (water).

Loss of barrier properties for TMM-4 was not observed, even after more than four months of immersion, showing an almost unchanged time dependence (Figure 12b). No significant differences were observed in the magnitude of the impedance (|Z|) in the high and medium frequency ranges. The durability of the coating could also be confirmed by the phase angle curves, which remained unchanged. After four months of immersion, the EIS measurements were discontinued. Although the thickness of the TMM-4 coating was only 1.4 μm, i.e., thin compared to other that of similar sol-gel coatings (usually more than five times thicker [27,40]), it offered superior corrosion protection.

The durability of the samples coated by TMM-6 was slightly lower than those coated by TMM-4, even though the coating was thicker (2.6 μm) (Figure 12c). The impedance values at the medium-high frequency varied greatly with longer copolymerization time. The small minimum of the phase angle noted between 10 Hz and 10 kHz may be related to the high degree of copolymerization which results in the formation of some pores in the coating structure [39]. Despite that, after three months of immersion, TMM-6 still exhibited similar values of impedance at low frequencies as at the beginning of immersion. However, after four months, the impedance values decreased at low frequencies. This reduction is related to the diffusion process of electrolyte through residual pores in the coating. This leads to a decrease in the magnitude of the impedance and a slight drop in the phase angle, both pronounced at low frequencies. However, the measured impedance value |Z_10 mHz_| of 3.8 MΩ cm^2^ is still quite large compared with that of uncoated aluminium alloy.

Comparison of all MMT coatings shows that TMM-4 exhibits a better long-lasting performance, which can be explained by the optimal copolymerization process and subsequent hydrolysis/condensation reactions. These results suggest the existence of an optimum copolymerization time that results in a dense hybrid structure with long-lasting, outstanding barrier properties.

## 4. Conclusions

The goal of this study was to identify the impact of copolymerization time on the structure and morphology of a hybrid material composed of acrylate monomers and a siloxane network. The effect of co-polymerisation time was thoroughly investigated at various steps, using different techniques. Several important conclusions can be drawn from the results:

- FTIR and DLS confirmed the formation of silica (Si–O–Si) domains from TEOS during hydrolysis/condensation reactions.
- FTIR, DLS and GPC confirmed that copolymerization between MMA and MAPTMS is time-dependent; the process was not complete even after 6 h.
- The structure of partially polymerised copolymers had a crucial effect on the subsequent hydrolysis/condensation reactions during formation of the organosilane matrix.
- The viscosity, and consequently the thickness, of the TMM coatings on AA7075-T6 also depended on the copolymerization time.
- The results of TG and DSC confirm that the optimal curing temperature is 166 °C.
- The FESEM/EDS established the presence of randomly spread silica-rich domains in the coating.
- The EIS revealed that the highest impedance was obtained with TMM-4 and TMM-6 coatings, confirming the importance of copolymerization time.
- Despite only a few-micrometre-thick coating, TMM-4 possessed high barrier properties and durability for more than four months in dilute Harrison’s solution.
- The TMM-4 coating exhibited the most substantial barrier properties, due to the more favourable copolymerised structure.

These results indicate that such an optimised coating can function as a pre-treatment for long-lasting corrosion protection of AA7075-T6.

## Figures and Tables

**Table 1:** Analysis, by gel permeation chromatography (GPC), of average molecular weight (M_w_), number average molecular weight (M_n_) and polydispersity index (PDI = M_w_/M_n_) for the MMA/MAPTMS polymers prior and after various copolymerization times (2, 4 and 6 h) and for TMM sols.

Sol	M_w_ [g/mol]	M_n_ [g/mol]	PDI
MMA/MAPTMS-prior	8724	6252	1.40
MMA/MAPTMS-2 h	81166	14881	5.08
MMA/MAPTMS-4 h	83333	16483	5.06
MMA/MAPTMS-6 h	147562	18737	7.88
TMM-2	79073	22215	3.56
TMM-4	79303	21149	3.75
TMM-6	81039	20097	4.03

**Table 2:** The density of the fluid (*γ*), kinematic (*ν*) and dynamic viscosity (*μ*) measured for TMM sols prepared after various copolymerization times (2, 4 and 6 h). Additionally, the estimated coating thickness on AA7075-T6 along the artificially made scribe evaluated by FESEM is added.

Sol	*γ* [g/mL]	*ν* [St]	*μ* [mPa s]	Coating Thickness [µm]
TMM-2	0.924563	2.68 ± 0.01	2.48	0.8 ± 0.2
TMM-4	0.924371	3.39 ± 0.01	3.31	1.4 ± 0.2
TMM-6	0.924616	4.21 ± 0.01	3.89	2.6 ± 0.3
